# The natural history of 21-hydroxylase autoantibodies in autoimmune Addison’s disease

**DOI:** 10.1530/EJE-20-1268

**Published:** 2021-01-29

**Authors:** Anette Boe Wolff, Lars Breivik, Karl Ove Hufthammer, Marianne Aardal Grytaas, Eirik Bratland, Eystein Sverre Husebye, Bergithe Eikeland Oftedal

**Affiliations:** 1 Department of Clinical Science, University of Bergen, Norway; 2 K.G. Jebsen Center for autoimmune disorders, University of Bergen, Norway; 3 Department of Medicine, University of Bergen, Norway; 4 Centre for Clinical Research, University of Bergen, Norway; 5 Department of Medical Genetics, Haukeland University Hospital, Bergen, Norway

## Abstract

**Background:**

The most common cause of primary adrenal failure (Addison’s disease) in the Western world is autoimmunity characterized by autoantibodies against the steroidogenic enzyme 21-hydroxylase (CYP21A2, 21OH). Detection of 21OH-autoantibodies is currently used for aetiological diagnosis, but how levels of 21OH-autoantibodies vary over time is not known.

**Setting:**

Samples from the national Norwegian Addison’s Registry and Biobank established in 1996 (*n* = 711). Multi-parameter modelling of the course of 21OH-autoantibody indices over time.

**Results:**

21OH-autoantibody positivity is remarkably stable, and >90% of the patients are still positive 30 years after diagnosis. Even though the antibody levels decline with disease duration, it is only rarely that this downturn reaches negativity. 21OH-autoantibody indices are affected by age at diagnosis, sex, type of Addison’s disease (isolated vs autoimmune polyendocrine syndrome type I or II) and HLA genotype.

**Conclusion:**

21OH-autoantibodies are reliable and robust markers for autoimmune Addison’s disease, linked to HLA risk genotype. However, a negative test in patients with long disease duration does not exclude autoimmune aetiology.

## Introduction

Acquired primary adrenal insufficiency (PAI) has multiple causes including autoimmunity, infections such as tuberculosis, genetic disorders, haemorrhage, and surgical removal. Sometimes the reason is obvious (surgery); in other cases, additional work-up is needed to ascertain the origin. Autoimmunity accounts for 75–96% of the cases in industrialised countries (1, 2, 3), defined by the presence of 21-hydroxylase autoantibodies (21OH-Abs) (4, 5, 6, 7, 8). Additional clues are the presence of organ-specific autoimmune comorbidities such as hypothyroidism, type 1 diabetes mellitus, and vitiligo.

21OH-Abs are present in 80–90% of patients with PAI in cross-sectional studies when known non-autoimmune causes have been excluded (9, 10), and is used in the diagnostic work up for this disorder (11). Data from a limited number of patients indicate that the frequency is higher shortly after diagnosis (>95%) (12), while it tends to fall with increasing disease duration reaching about 50% after 20 years (7, 12). Thus, if 21OH-Abs are assayed many years after diagnosis, a negative result does not exclude autoimmunity. Conversely, 21OH-Abs can be present in individuals with completely normal adrenal function, where their presence signals a future risk of developing overt PAI (13, 14). Both environmental and genetic factors are involved in the pathogenesis, and certain HLA alleles confer a high risk of developing the disease (15, 16, 17, 18).

We have limited information on the natural history of 21OH-Abs in Addison’s disease and how many become autoantibody negative over time. We hypothesise that the diagnostic value of 21OH-Abs declines with time and that patients might have autoimmune Addison’s disease despite the lack of autoantibodies. By using a national registry and biobank established in 1996 with serial samples including the majority of Norwegian patients with Addison’s disease, we aimed to investigate the robustness of the 21OH-Abs assay and its clinical value in defining autoimmune PAI.

## Methods

### Patients

The study was approved by the Regional Committees for Medical and Health Research Ethics (project nos. 2013/1504 and 2018/1417), with written informed consent obtained from each participant after full explanation of the purpose and nature of all procedures used. The National Norwegian Addison Registry (ROAS) collates clinical information, demographics, and biological samples from patients with PAI. 21OH-Abs status is assayed in all patient samples when they are included in the registry and every time a successive sample is included in the biobank. The samples are analysed with the same 21OH-Abs assay method in the same laboratory. Those with a positive result are classified as autoimmune PAI. Presence of autoimmune polyendocrine syndrome type I (APS-I) is defined by two of the following three components PAI, hypoparathyroidism, and chronic mucocutaneous candidiasis, type I interferon antibodies and disease-causing variants in the *Autoimmune Regulator* (*AIRE*) gene. All samples in the registry are screened for interferon omega antibodies to exclude undiagnosed APS-I. APS-II is categorised as PAI concomitantly with type 1 diabetes and/or autoimmune thyroid disease. Details regarding clinical criteria for PAI and comorbidities are given in previous publications on the Norwegian PAI cohort and registry (20).

Patients in ROAS without 21OH-Abs are screened for other causes, usually with imaging of the adrenals in adult patients (to reveal signs of infection, tumour and haemorrhage), and genetic screening for causes such as adrenal hypoplasia congenita (DAX1-mutations) (21) and adrenoleukodystrophy (accumulation of very-long chain fatty acids and mutations in *ABCD1*) (22). Those without known cause are classified as idiopathic, but probably autoimmune PAI. Altogether 711 patients with autoimmune and idiopathic PAI were included, and their characteristics are reported in Table 1. Exclusion criteria were PAI-patients with known non-autoimmune causes.

**Table 1:** Characteristics of the 711 included patients with autoimmune Addison’s disease. Data are presented as mean ± s.d.

	**PAI**	**APS-II**	APS-I with PAI*
Patients, *n*	305	372	34
Frequency of females (%)	50.3	72.0	41.2
Age at diagnosis (years)	33.1 ± 15.4	36.6 ± 14.8	15.7 ± 9.6
Time between diagnosis and first sample (years)	11.5 ± 13.5	11.2 ± 12.7	15.0 ± 15.3
21OH-Abs index	577± 356	620 ± 319	317 ± 228
Positivity for 21OH-Abs (%)	88.2	93.8	85.3
Males	89.4%	93.2%	
Females	87.6%	93.6%	

APS, autoimmune polyendocrine syndrome; PAI, primary adrenocortical failure (isolated).

**Figure 1:**
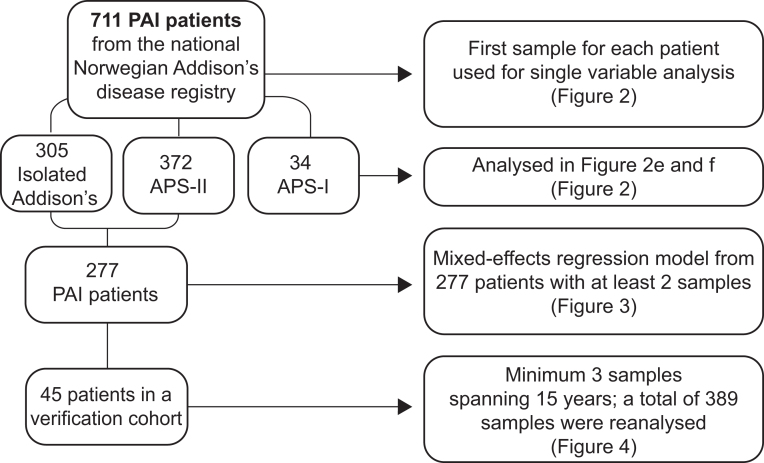
Flow chart describing the patients in this study.

In addition, we re-assayed 21OH-Abs in serial samples (total number of samples, 389, range 3–15 samples from each patient, median 9) from 45 of the 711 patients. This ‘verification cohort’ included patients who were recruited to the registry between 1996 and 2001 with a minimum of three samples spanning at least 15 years and with at least one sample positive for 21OH-Abs. Samples from a particular patient were analysed on the same plate to avoid plate-to-plate fluctuations in indices of 21OH-Abs. Patients with APS-I verified by sequencing of *AIRE* (23) and diagnosed with PAI were included in the registry-based segment of the study (see below). A flow chart on the included patients and samples in this study is shown in Fig. 1.

### Assay of 21OH-Abs

An in-house radio-ligand binding assay was employed to detect 21OH-Abs as described previously (24). A positive (index 1000) and negative (index 0) control was used to calculate the 21OH-Abs index. The threshold for positivity has been set by assaying 150 healthy controls and calculating the mean 21OH-Abs index + 3 s.d. This assay, including the threshold for positivity and variance numbers, has further been tested and verified by comparing with other European laboratories in the Euradrenal Consortium (10). The samples are usually analysed within a month of sampling. All samples are stored at −80°C.

### Human leukocyte antigen (HLA) determination and risk of developing autoimmune PAI

Genotypes for HLA-DRB1 and HLA-DQB1 were analysed with a PCR-based sequence-specific oligonucleotide probe system at four-digit resolution or imputed from the Global Screening Array chip (15, 18). The HLA-DQA1 alleles and the HLA-DRB1-DQA1-DQB1 haplotypes were deduced based on known patterns of linkage disequilibrium in the Norwegian population. The HLA-DRB1-DQA1-DQB1 genotypes were stratified into three risk categories according to previously reported risk HLA variants for autoimmune PAI (12, 15) (Table 2), also supported by data from a recent genome-wide association study on autoimmune PAI (18).

**Table 2:** Classification of HLA genotypes and applied risk class.

**Risk class** (this study)	**Allele 1** (HLA-DRB1-DQA1-DQB1)	**Allele 2** (HLA-DRB1-DQA1-DQB1)	**RISK category**
1	*0301-*0501-*0201	*0404-*0301-*0302	Very high
1	*0404-*0301-*0302	*0404-*0301-*0302	High
1	*0301-*0501-*0201	*0301-*0501-*0201	High
2	*0301-*0501-*0201	Any other DRB1 allele	Intermediate
3	*0404-*0301-*0302	Any other DRB1 allele	Low
3	Intermediate*	Intermediate *	Low
3	Intermediate*	Low*	Very low
3	Low*	Low*	Very low

* Intermediate: The combination of any of the following HLA-DRB1-DQA1-DQB1 haplotypes: *1401-*0101-*0503, *15-*0102-*0602/*0611, *07-*0201-*0303, *0401-*0301-*0301, *0801-*0401-*04, *11-*0501 -*0301and/or *12-*0501-*0301.

*Low risk: Combination of the HLA-types in the ‘Intermediate risk’ category or the combination of any of the following HLA-DRB1-DQA1-DQB1 haplotypes:*01/*10-*0101-*0501, *1301-*0103-*0603, *1302-*0201-*0604, and/or *07-*0201-*0201.

HLA-data from 285 of the included PAI patients (isolated PAI and APS-II) with disease duration < 5 years at sampling were available. The genotypes were categorised into the three groups according to the estimated risk these variants confer for developing PAI ; *n* = 70, low risk, *n* = 115, intermediate risk, and *n* = 100, high risk (Table 2). In the mixed effects model explained below where all patients in the registry with >2 samples were included, there were 80, 91 and 107 individuals in each of the risk groups, respectively.

### Statistical analysis

#### Impact on individual parameters for 21OH-Abs frequency and index

**Figure 2:**
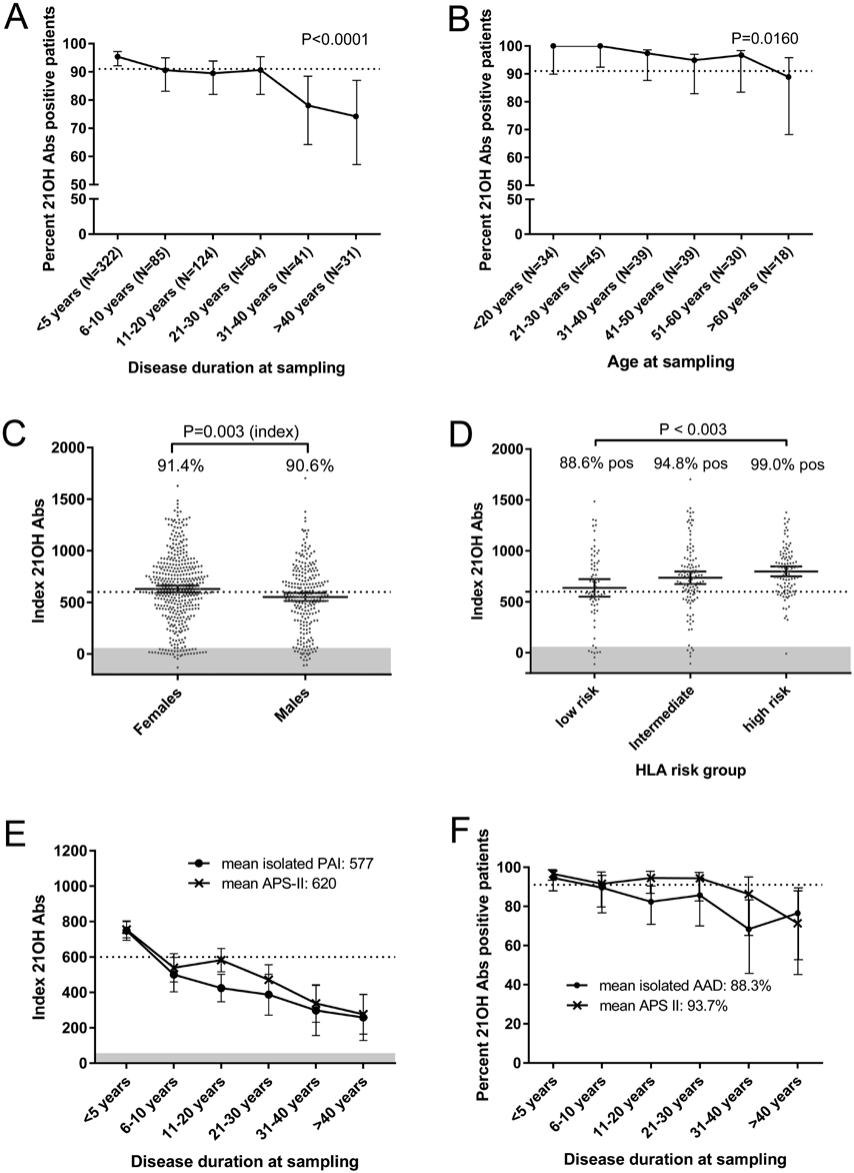
Individual variables’ impact on 21OH-Abs frequency and indices (A) Percentage of isolated PAI and APS-II patients positive for antibodies against 21OH (21OH-Abs) separated in groups according to disease duration at sampling. The statistical test was done with logistic regression analysis. (B) Percentage of isolated PAI and APS-II patients positive for 21OH-Abs sampled within 1 year of diagnosis in relation to age at diagnosis. Statistical analysis was done with logistic regression analysis. (C) 21OH-Abs-indicies in males and females. Percentage positive for each sex is shown inside the corresponding box. Statistical test for indices between the groups was achieved by a parametric t-test while statistical differences between the frequencies were done by a Pearson’s chi test. (D) 21OH-Abs indices stratified by HLA-risk groups given in Table 2 and percentage positives in each group. Statistical differences were calculated by ANOVA and Tukey’s test. (E) 21OH-Abs-indices stratified into phenotypic groups and disease duration at sampling; isolated PAI (black) and APS-II (red). (F) Percent positive for 21OH-Abs stratified to disease duration at sampling; isolated PAI (black) and APS-II (red). The dashed line (A, B and F) represents the mean positivity for autoantibodies against 21OH-Abs in the whole isolated, AAD autoimmune PAI and APS-II cohort. The dashed line (C and E) represents the mean indices of 21OH-Abs. The shaded area (C–E) in the lower part of the graph represent where the test gives negative results (sets the threshold for positivity). 95% CI is shown by vertical bars.

Statistical tests on frequencies of groups were performed with logistic regression analysis (Fig. 2A and  B). A parametric Student’s *t*-test was employed to compare sex-differences regarding 21OH-Abs indices, and a Pearson’s chi test to compare 21OH-Abs frequencies (Fig. 2C). ANOVA was used for comparing multiple groups, and Tukey’s multiple comparisons test was applied to compare statistical differences between groups (Fig. 2D).

#### Mixed effects regression model

To fully exploit our longitudinal sampling of patients with autoimmune PAI, and because autoantibodies against 21OH are assayed in every sample recruited to ROAS, we included all samples from PAI patients with at least two measurements, excluding APS-I (*n* = 711) to generate a mixed-effects regression model of the 21OH-Abs course over time. We adjusted for disease duration (sampling date from date of diagnosis), age, sex, type of PAI (isolated Addison’s disease vs APS-II), and HLA-risk genotype (low risk, intermediate risk, high risk) as covariates.

To model the trajectories of 21OH-Abs over time, we fitted a mixed-effects regression model. The explanatory variables were disease duration (years from diagnosis), age at diagnosis, sex, HLA risk category (three levels), and APS type (isolated Addison’s disease vs APS-II). Patients with APS-I were excluded in this model due to low numbers and monogenic cause.

Initial visualisations of the data indicated a non-linear association with years from diagnosis, so this variable was included as second-degree orthogonal polynomial. Random effects were included for the coefficients of the polynomial (including the intercept). This takes into account the dependence between samples from the same individual, and it allows each patient to have their own second-degree curve. However, unlike models where one fits second-degree curves for each patient separately, when estimating the curves in the mixed-effect model, information is ‘borrowed’ from the whole population. This allowed us to reliably estimate second-degrees curves also for patients with very few samples. For this model, we included data from all patients who had at least two available samples (*n* = 227).

High 21OH-Abs indices seemed to have larger variance. In the regression model, we therefore modelled the variance as a linear function of the predicted values. Examination of residuals plots indicated that the resulting model fitted the data well.

The data was analysed with Graphpad Prism 7 and R version 4.0.2 (25). 95% confidence intervals were calculated with a fraction of total analysis using the Wilson/Brown method. The mixed-effects model was fitted with the R package ‘nlme’ version 3.1-149 (26). *P* values ≤ 0.05 were characterised as statistically significant (Table 3).

**Table 3:** Multivariable mixed-effects longitudinal regression model for 21OH-Abs (*n* = 1287 observations from 277 individuals).

**Variable**	**Coeff.**	**95% CI**	*P***-value**
Time from diagnosis (nonlinear)	–	–	< 0.001
Age at diagnosis (years)	−2.2	−4.2 to −0.3	0.03
Sex, female	117	55 to 178	<0.001
HLA risk group			
Low	0	–	–
Intermediate	99	32 to 167	0.004
High	69	19 to 119	0.007
APS-II, yes	75	16 to 124	0.01

21OH-Abs, 21-hydroxylase autoantibodies; APS-II, Autoimmune polyendocrine syndrome; 2Coeff, regression coefficient.

## Results

### Description of the patient cohort

Altogether 711 patients were included, 305 with isolated PAI, 34 with APS-I, and 372 with APS-II. The sex ratio was equal among isolated PAI (50.3% females), and APS-I (41.2% females), while females dominated the APS-II group (72.0%). Mean age at diagnosis was 33.1 (s.d. 15.4), 15.7 (s.d. 9.6) and 36.6 (s.d. 14.8) years for isolated PAI, APS-I and APS-II, respectively. The mean time between diagnosis and the first available blood sample varied between 11.2 and 15.7 years for the disease groups. Positivity of 21OH-Abs was >85% in all three groups; the mean 21OH-Abs indices were higher for isolated PAI (mean: 577) and APS-II (620), than for APS-I (317). The results are summarised in Table 1.

### 21OH-Abs in the first available sample according to age, disease duration, sex, HLA risk and Addison’s disease type

Intriguingly, >90% of PAI-patients with diagnosis up to at least 60 years of age had 21OH-Abs, and patients retained these autoantibodies up to 30 years after diagnosis (Fig. 2A and  B). For patients with disease duration that exceeds 30 years, the 21OH-Abs frequency fell to ~75% in this ‘one-variable’-analysis (Fig. 2A, *P* < 0.0001), showing a trend towards a decrease of 21OH levels over time.

We further investigated whether sex, HLA genotype, and type of PAI influenced the 21OH-Abs status as single variables. To this end, we categorised the 21OH-Abs indices from the first sample from each patient according to presence of (i) an autoimmune syndrome or not (isolated PAI, APS-I, and APS-II), (ii) sex, and (iii) HLA risk group, looking at one parameter at a time. For HLA, only patients with disease duration from diagnosis to sampling <5 years were included. While there was a difference in indices of 21OH-Abs between females and males (*P* = 0.003, 95% CI (26.1 to 130)), no difference in 21OH-Abs frequencies between females (91.4%) and males (90.6%) was detected (Fig. 2C). Intriguingly, we found that presence of 21OH-Abs correlates with HLA-risk genotype. In patients with low-risk HLA-types, 88.6% had 21OH-Abs (mean index 637), as opposed to 94.8% in the intermediate group (mean index 736) and 99.0% in the high-risk group (mean index 798) (overall ANOVA *P* < 0.00002) (Fig. 2D).

Looking at the three patient categories of (i) isolated PAI, (ii) APS-I and (iii) APS-II, we discovered several interesting patterns (Fig. 2E and  F) (APS-I not shown). First, there was a trend that patients with APS-I had lower levels of 21OH-Abs until 31–40 years, but then indices increased compared to patients with isolated PAI. The pattern of 21OH-Abs levels in isolated PAI and APS-II were similar at very young ages (<10 years), starting at indices about 700–800 with approximately 95% of samples positive. At older disease durations, APS-II patients tended to have both higher frequencies and indices of 21OH-Abs than the isolated, autoimmune PAI group. Both groups had declining frequencies and indices over time, although indices were still positive in the area of 200–300 and at frequencies of 70–80%, 40 years after diagnosis.

### Unified model of 21OH-Abs indices shows a decline with disease duration, and dependence on sex, type of Addison’s disease and HLA-risk group

**Figure 3:**
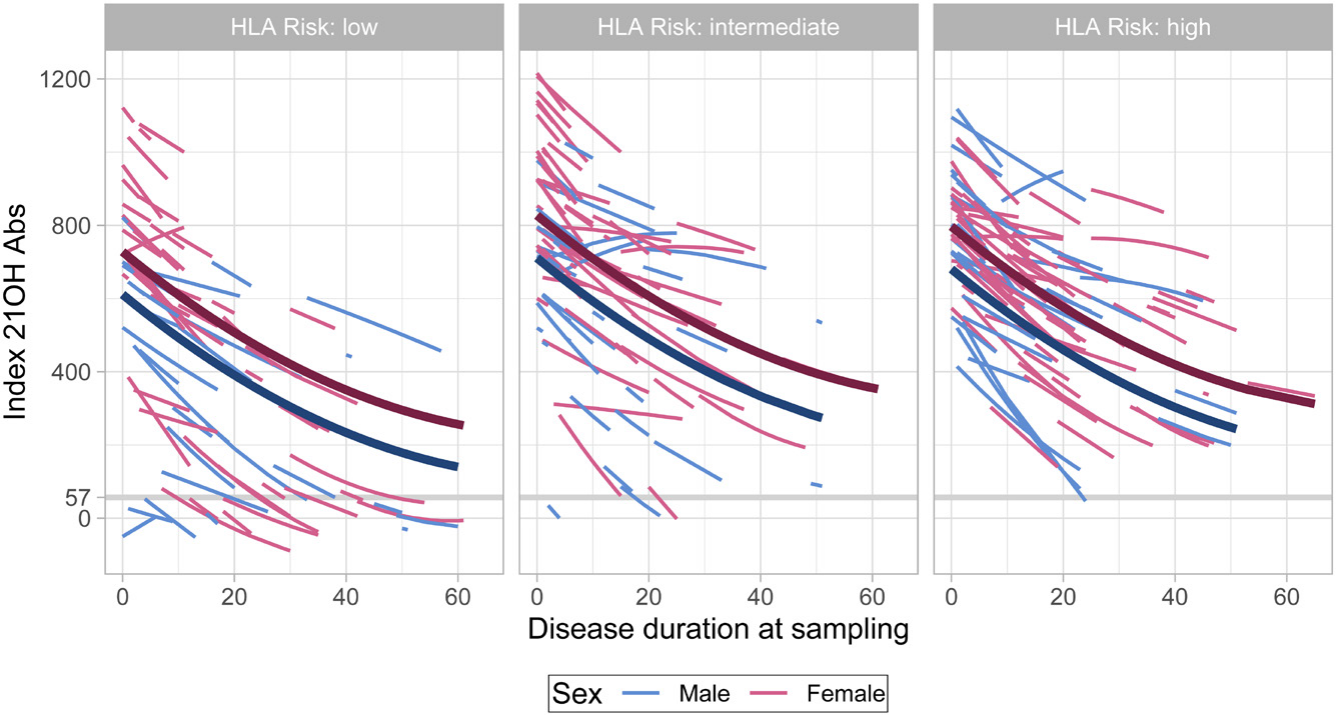
Estimated individual trajectories from mixed-effects longitudinal regression model for 21OH-Abs (*n* = 1287 observations from 277 individuals). The lines corresponds to individual patients. Includes data from patients with at least two samples. The thick lines show the predicted trajectory for a ‘typical’ patient (all random effects set to 0), that is a 40-year-old female with APS-II.

The unified model shows that indeed the 21OH-Abs indices contract over time, but it seldom reaches values below the threshold for positivity, at least not in those with intermediate and high risk HLA genotypes (Fig. 3). The modelled indices are higher in females than males throughout the time span in all HLA categories. Looking at HLA risk groups, the 21OH-Abs is constantly lower in the low risk group compared to intermediate and high-risk group (Fig. 3). The strongest association to 21OH-Abs was disease duration, followed by HLA risk group, and then sex (Table 3).

**Figure 4:**
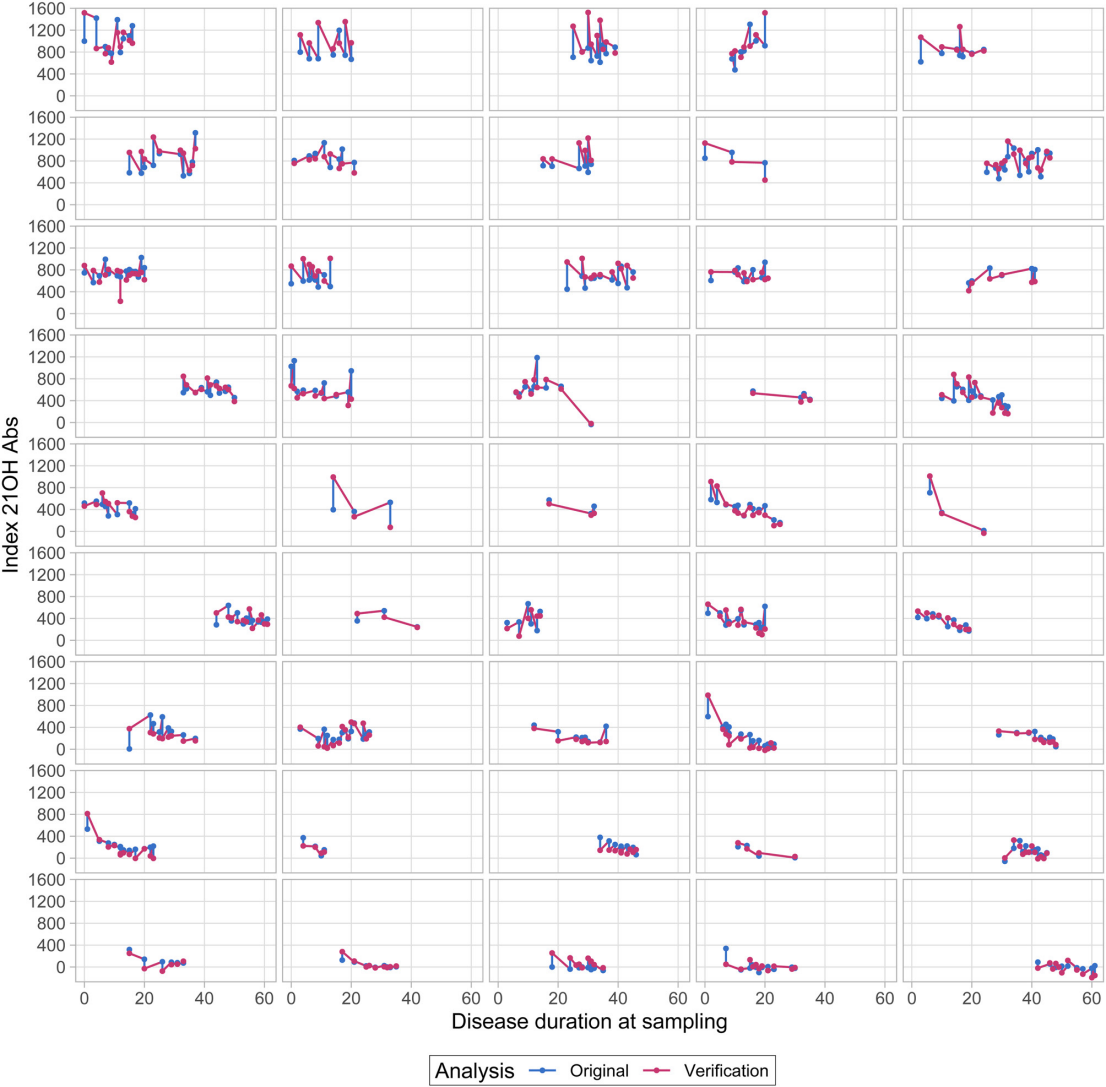
Course of 21OH-Abs in the 45 patients from the verification study (n = 389 samples) assayed at inclusion in the registry and in the verification study.

To verify the registry results of declining levels of 21OH-Abs due to long disease duration (Figs 2A and  3), we reinvestigated longitudinal samples in 45 patients, running a total of 389 samples from the same patients in the same assay to exclude inter-assay variation (Fig. 4). We found that some patients had antibody levels that decreased very early after diagnosis, sometimes becoming negative. Others were stable at a high level over an extended period of time, while yet others revealed a pattern with large fluctuations in indices. Importantly, the curves have similar patterns for each patient when comparing data from the registry and the verification study (Fig. 4 and Supplementary Fig. 2, see section on supplementary materials given at the end of this article). There are, however, some deviations between the two analysis points, resulting in a fan-shaped deviation graph (Supplementary Fig. 1). This shows that the analytical variation is larger for higher indices, and lower for the points around the threshold for positivity. The stability of the assay, the independence of the person performing the assay and the adequate stable results after repeated freezing and thawing of samples is highlighted by the good correlation between the biobank-recorded value and our verification study.

## Discussion

We have investigated the robustness and clinical value of analyzing autoantibodies against 21OH in patients with PAI, APS-I, and APS-II and how they persist over the time course of the disease. 21OH-Abs have been analysed in samples from a national registry with over 700 patients, with samples spanning more than 20 years for several of them. The 21OH-Abs have also been analysed in the context of sex, HLA risk genotypes, and underlying genetic mutations (APS-I). In addition, we validated our finding in a subgroup of patients by running longitudinal samples. Our main finding is that the 21OH-Abs are remarkable stable, even up to 30 years after diagnosis.

When only considering the first available sample from each patient and looking at each of the factors individually (duration between diagnosis and sampling, age, sex, presence of an APS and HLA risk category), we found that all factors statistically contribute to the autoantibody indices, which declined during disease duration. As APS-I is a rare syndrome, only 34 patients could be included, and the results for this group should be interpreted with caution. Due to their low number and the monogenic form of disease these samples were excluded from the rest of the study. Making a unified model for the course of 21OH-Abs using all available samples from the start of ROAS in 1996, we show that all included co-factors affected the 21OH-Abs level.

A decline in autoantibody index in relation to age or disease duration could prevent correct diagnosis of autoimmune disorders. However, even though we found such a decline in 21OH-Abs positivity, more than 90% of the patients still had a positive 21OH-Abs index 30 years after diagnosis. This is in agreement with a previous study from this cohort of patients (12) and verifies that 21OH-Abs are excellent biomarkers for PAI. It also suggests that autoantibodies are generated for decades after the diagnosis is made. Although beyond the scope of this study, it raises the question of which factors are still priming the autoimmune reaction leading to production of these autoantibodies. One possibility is the existence of a small functional adrenal that keeps triggering the response, consistent with our results recent results that about 30% of patients have some residual adrenal function. from a newly published study showing presence of adrenal rest, even after decades of clinical disease (27).

We were further intrigued by the differences in 21OH-Abs positivity related to HLA, from 88% in the ‘low risk HLA-group’ to >99% in the ‘high HLA risk group’. HLA is an immunological determinant as it is the molecule that presents external or internal peptides to T cells. The HLA genotype works as an individuals’ fingerprint which determines what peptides an immune reaction can be mounted against. Indeed, PAI is a highly heritable disease (28), and a large component of the heritability relies on HLA-genotypes (12, 15, 16, 17, 29, 30, 31, 32, 33). Consistent with previous studies, a recent GWAS concluded that the risk was dominated by HLA-DQB1*02:01 (part of the DR3-DQ2 haplotype, OR = 5.71) and HLA-DQB1*03:02 (part of the DR4-DQ8 haplotype, OR = 5.57) (18). Our findings of less 21OH-Abs in the low-risk HLA group opens for speculation if other hitherto unknown autoantigens are at work. If such autoantibodies exist, they would be valuable additional diagnostic tools and might shed light on the pathogenic actions underlying adrenal autoimmunity.

In conclusion, we have here shown that 21OH-Abs positivity is remarkably stable over time, providing a robust biomarker to establish the presence of autoimmune PAI. Even if the autoantibody titre does decrease during long duration time, >90% of patients still retain 21OH-Abs 30 years after diagnosis.

## Supplementary Material

Figure 1. Total deviations between 21OH-Abs indices from the registry and the verification study with logistic regression analysis. The graph shows the relation between the 21OH-abs index values generated consecutively as samples were biobanked (registry) and the index found on retesting (verification study), one data point per patient. The y-axis is the original index minus the new index for a given sample; the x-axis represents the mean of the original and new index. subtracted by the new value (y-axis) and the mean indices on the x-axis.

Figure 2. Deviations between 21OH-Abs indices from the registry and the verification study shown for each patient. The graph shows the relation between the 21OH-abs index values generated consecutively as samples were accepted to the biobank (registry) and the index found on retesting (verification study), in patients were several samples were available.

## Declaration of interest

The authors declare that there is no conflict of interest that could be perceived as prejudicing the impartiality of the research reported.

## Funding

The study was funded by the Western Norway Regional Health Authority, the Norwegian Research Council and the K.G. Jebsen Center for autoimmune disorders.
