# Supplementary figures and images for: The natural history of 21-hydroxylase autoantibodies in autoimmune Addison’s disease
Source: Eur J Endocrinol. 2021 Jan 29;184(4):607–15. doi: 10.1530/EJE-20-1268 (PMC8052519; doi:10.1530/EJE-20-1268)

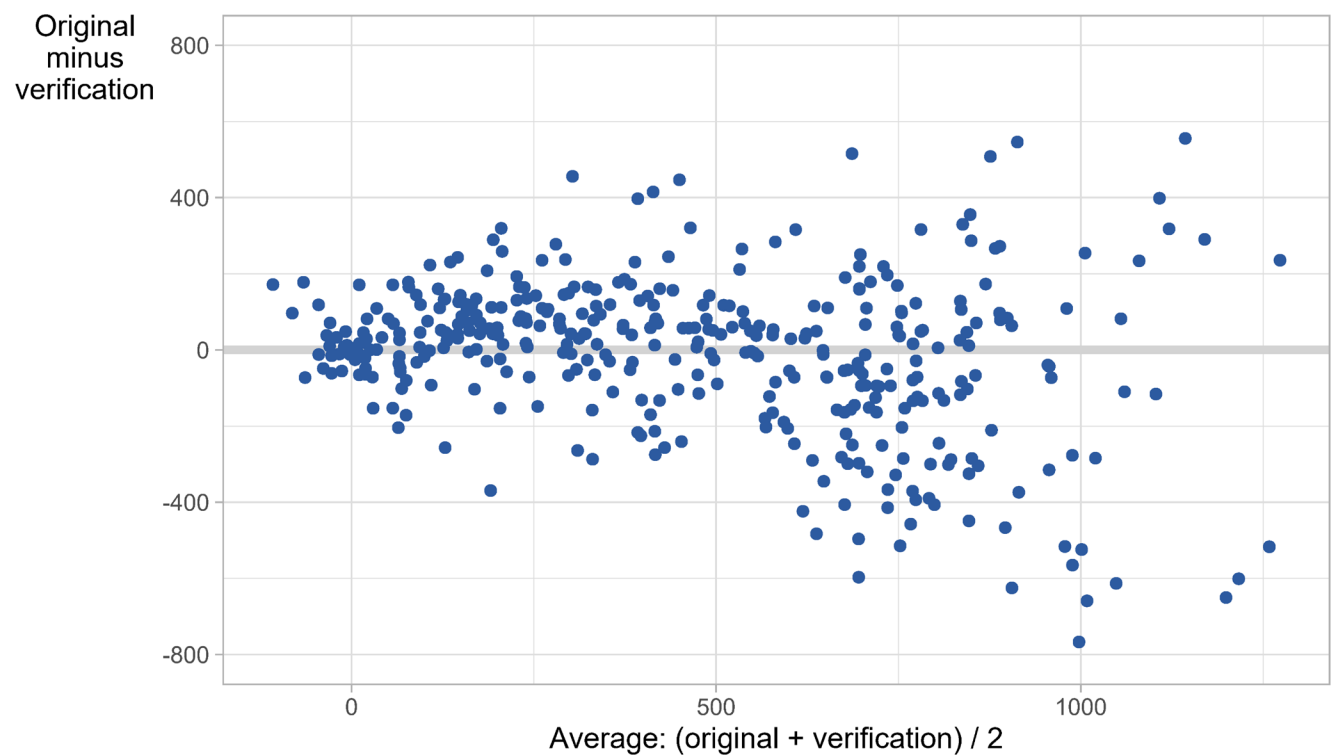

Supplement: Figure 1. Total deviations between 21OH-Abs indices from the registry and the verification study with logistic regression analysis. The graph shows the relation between the 21OH-abs index values generated consecutively as samples were biobanked (registry) and the index found on retesting (verificati [file supplementary_figure_1.pdf]

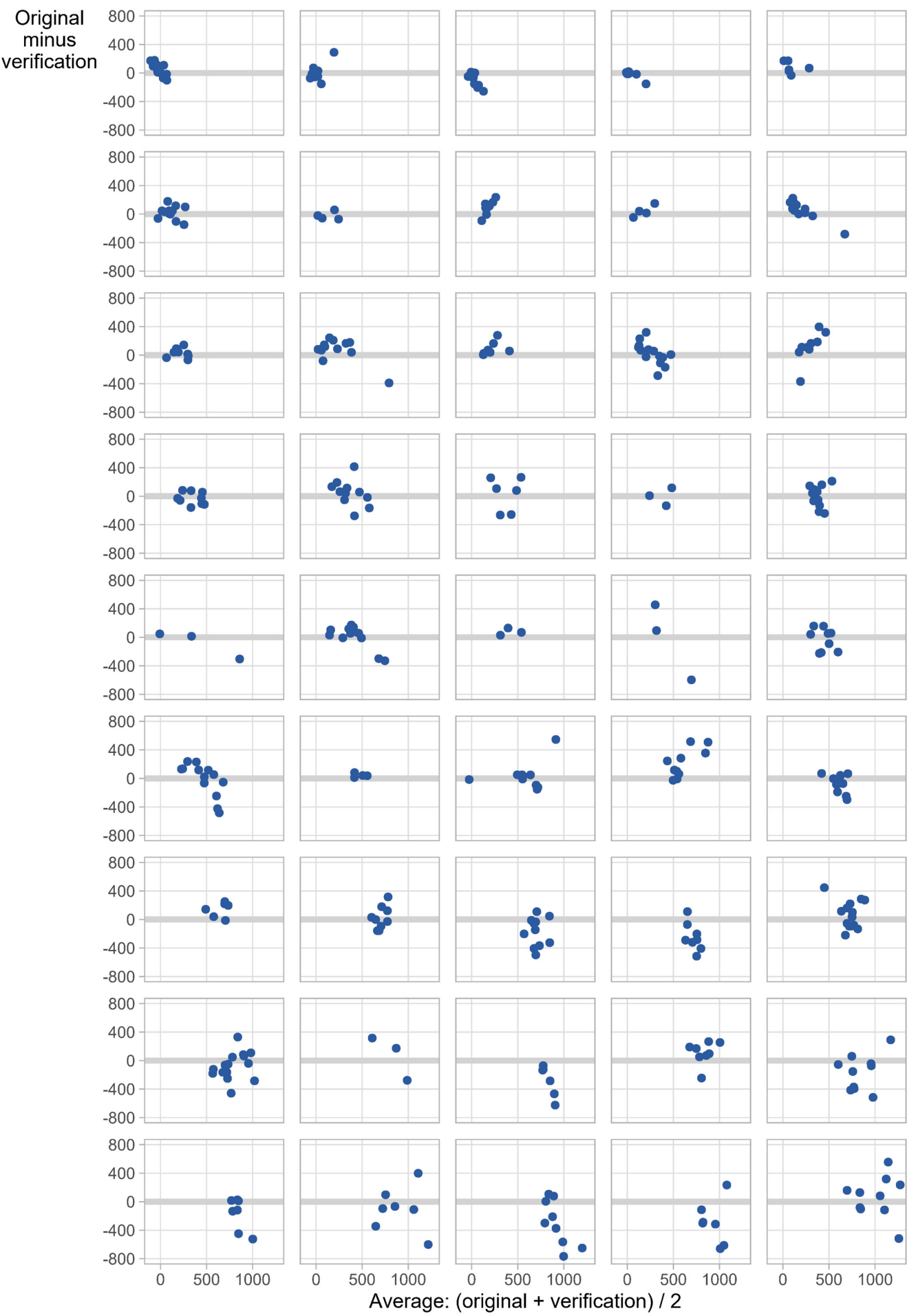

Supplement: Figure 2. Deviations between 21OH-Abs indices from the registry and the verification study shown for each patient. The graph shows the relation between the 21OH-abs index values generated consecutively as samples were accepted to the biobank (registry) and the index found on retesting (verification  [file supplementary_figure_2.pdf]
